# Factors Associated with Extended-Spectrum β-Lactamases and Carbapenem-Resistant *Klebsiella pneumoniae* Bloodstream Infections: A Five-Year Retrospective Study

**DOI:** 10.3390/pathogens12111277

**Published:** 2023-10-25

**Authors:** Andreas G. Tofarides, Panagiotis Dimitriou, Georgios K. Nikolopoulos, Dimitrios Rogkas, Christina Flourou, Elina Khattab, Diamanto Kasapi, Chara Azina, Eirini Christaki

**Affiliations:** 1 Department of Internal Medicine, Nicosia General Hospital, 2029 Nicosia, Cyprus; panagiwtis_dtriou@hotmail.com (P.D.); christinafl@hotmail.gr (C.F.); khattab_elina@outlook.com (E.K.); diamanto_kasapi@hotmail.com (D.K.); chara.azina@hotmail.com (C.A.); 2 1st Division of Internal Medicine & Infectious Diseases Unit, University Hospital of Ioannina, Faculty of Medicine, University of Ioannina, 45500 Ioannina, Greece; md06569@uoi.gr; 3 Medical School, University of Cyprus, 2029 Nicosia, Cyprus; gknikolopoulos@gmail.com

**Keywords:** *Klebsiella pneumoniae*, bloodstream infection, nosocomial infection, antimicrobial resistance, extended-spectrum b-lactamases, carbapenem resistance

## Abstract

*Klebsiella pneumoniae* is one of the leading causes of nosocomial infections. It has been estimated that nosocomial infection by *Klebsiella pneumoniae* comprises 3–8% of all nosocomial infections. *Klebsiella pneumoniae* bloodstream infections (BSIs) occur worldwide with varying mortality. Resistant strains, like those producing extended-spectrum beta-lactamases (ESBL) and carbapenemases, are becoming increasingly common, especially in hospital settings, posing therapeutic challenges. In this article, we aimed to study the epidemiology and risk factors of BSIs due to resistant *Klebsiella pneumoniae* strains in the period 1 January 2014–31 December 2018 at the Nicosia General Hospital, the largest tertiary hospital in Cyprus. Data on demographics, co-morbidities, prior hospitalization, prior intensive care unit (ICU) admission, previous antimicrobial use, nosocomial acquisition of the infection, the presence of a prosthetic device or surgery, and the primary site of infection were retrospectively recorded. Associations between the detection of ESBL *Klebsiella pneumoniae* BSIs and factors/covariates were examined using logistic regression. This study involved 175 patients with BSI caused by *Klebsiella pneumoniae*. Of these, 61 BSIs were caused by ESBL strains, 101 by non-ESBL, and 13 by carbapenem-resistant (CR) strains. In univariable analyses, age, sex, heart disease, antimicrobial use during current admission, previous hospitalization (ward or ICU), and primary BSI were associated with the presence of an ESBL strain. Antibiotic use during current admission and heart disease remained statistically significantly associated with ESBL *Klebsiella pneumoniae* BSI in multivariable models. Antibiotic use during current admission, respiratory infection, and a recent history of surgery were more prevalent among CR *Klebsiella pneumoniae* BSI patients than among non-CR *Klebsiella pneumoniae* BSI patients. Our study showed that recent antimicrobial use and heart disease were associated with BSI due to ESBL-producing *Klebsiella pneumoniae*. This finding could inform clinical practice in hospital settings.

## 1. Introduction

*Klebsiella pneumoniae* belongs to the enterobacteriaceae genus [1]. Among the pathogenic species (*Klebsiella pneumoniae*, Klebsiella ozaenae, Klebsiella rhinoscleromatis, and Klebsiella oxytoca), *Klebsiella pneumoniae* is the most common and clinically significant. It is also one of the leading causes of nosocomial infections, showing a prevalence varying up to 10% [2] and causing urinary tract, respiratory system, or bloodstream infections (BSIs)**.** In addition, Κlebsiella pneumoniae causes wound infections, otitis, sinusitis, and, less often, central nervous system (CNS) infections [3,4].

According to the annual report by the European Centre for Disease Prevention and Control (ECDC), which includes data from the European Antimicrobial Resistance Surveillance Network (EARS-NET), in 2020, 30 European Union (EU)/European Economic Area (EEA) countries reported 40,075 isolates of *Klebsiella pneumoniae*. Of these, more than a third (38%) were resistant to at least one of the antimicrobial groups under surveillance (i.e., fluoroquinolones, third-generation cephalosporins, aminoglycosides, and carbapenems). In 2020, the highest EU/EEA population-weighted mean resistance percentage was reported for third-generation cephalosporins (33.9%), followed by fluoroquinolones (33.8%), aminoglycosides (23.7%), and carbapenems (10%) [5]. Potential risk factors for infections caused by *Klebsiella pneumoniae*-resistant strains include age, gender, prior recent hospitalization (within 3 months), history of intensive care unit (ICU) admission, the presence of medical devices, hemodialysis, recent surgery, active cancer under chemotherapy, solid organ transplantation, a central venous catheter (CVC), and the prior use of antibiotics [6,7]. *Klebsiella pneumoniae* strains, which produce extended-spectrum beta-lactamases (ESBLs) and carbapenemases, have a worldwide distribution, albeit with regional differences. *Klebsiella pneumoniae* strains producing ESBL are resistant to most beta-lactam antibiotics including penicillins, cephalosporins, and the monobactam aztreonam. Furthermore, *Klebsiella pneumoniae* strains that produce carbapenemases have also spread rapidly and are the cause of multidrug-resistant infections (MDR) in many countries [8,9,10]. 

*Klebsiella pneumoniae* strains, resistant to broad-spectrum antibiotics, cause severe infections, especially in immunocompromised patients, those with prior surgery, those with multiple comorbidities, or those who have a permanent bladder catheter [11,12,13,14,15]. Particularly, carbapenem-resistant (CR) *Klebsiella pneumoniae* is associated with an approximately 42% mortality rate [16] and is one of the most life-threatening pathogens that causes invasive infections. In a recent systematic review of 157 studies [17], the overall mortality rates were 17% at day 7, 24% at day 14, 29% at day 30, 34% at day 90, and 29% in hospital. However, with antimicrobial resistance rates increasing annually, accurate estimates of mortality have been insufficient [17]. 

This five-year retrospective study aimed to study the epidemiology and risk factors of BSIs due to resistant *Klebsiella pneumoniae* strains between 1 January 2014 and 31 December 2018 at the Nicosia General Hospital, the largest tertiary hospital in the Republic of Cyprus.

## 2. Materials and Methods

This study involved patients with BSI due to *Klebsiella pneumoniae*, who were hospitalized at the Nicosia General Hospital between January 2014 and December 2018. Data were collected from electronic and paper patient records, the hospital Microbiology Laboratory’s database, and WHONET 5.6, which is a software developed by the World Health Organization (WHO). Data on demographics (age and gender) and characteristics such as co-morbidities (diabetes mellitus, pulmonary disease, heart disease, malignancy and related treatment, autoimmune disease, hepatic disease, kidney disease, alcohol use, and smoking), prior hospitalization, prior ICU admission, previous antimicrobial use, nosocomial acquisition of the infection, type of infection (urinary infection, hospital-associated pneumonia, abdominal infection), previous surgery in the last three months, the presence of prosthetic device, and primary site of infection were retrospectively recorded. All data were stored in electronic password-encrypted databases. This study was approved by the Cyprus National Bioethics Committee (date 13 June 2019, number: 2019.01.108).

The analysis focused on: (a) the epidemiology of multidrug-resistant (MDR) *Klebsiella pneumoniae* infections and (b) the association between predisposing factors and BSI caused by multidrug-resistant strains of *Klebsiella pneumoniae.* Chi-squared tests and t-tests were used to explore associations between ESBL or CR *Klebsiella pneumoniae* BSIs and factors/covariates. Associations between ESBL *Klebsiella pneumoniae* BSIs and risk factors were examined using logistic regression (univariable and multivariable models). All variables that were statistically significant (*p* < 0.20) in the univariable analyses, had clinical importance, or were considered important according to the published literature, were used in multivariable modeling. More specifically, multiple logistic regression was performed initially using statistically significant variables from the univariable models. Subsequently, the significant variables were checked simultaneously with all the other variables, one by one. The final analysis consisted of two multivariable models: (i) Model A, which included the final statistically significant variables from the aforementioned process, and (ii) Model B, which included variables that were considered the most important both in statistical terms and based on what has been reported in the literature (sex, antibiotic use during hospitalization, heart disease, and previous hospitalization in a regular ward or ICU). 

The statistical analyses were performed in *R* (R studio, version 3.6.1). Analyses code (R script) is available as a Supplementary Material.

## 3. Results

The analysis included 175 patients (33.7% males) who were hospitalized for bloodstream infection due to *Klebsiella pneumoniae* between January 2014 and December 2018. Of these, 61 BSIs were caused by ESBL strains (34.9%), 101 by non-ESBL strains, and 13 by CR strains (7.4%).

The comparison of patients’ variables between those with BSI due to ESBL strains and those with BSI due to non-ESBL strains using chi-squared tests and t-tests is shown in Table 1. The results from the logistic regression models are shown in Table 2. According to the univariable logistic regression analyses, age (Odds Ratio (OR) 1.02, 95% Confidence Interval (Cl): 1.00–1.04), sex (male) (OR 2.18, 95% Cl 1.12–4.28), heart disease (OR 4.04, 95% Cl: 2.08–8.06), antimicrobial use during current admission (OR 4.05, 95% Cl: 2.04–8.39), previous hospitalization (OR 1.92, 95% Cl: 0.99–3.81), previous hospitalization in the ICU (OR 3.29, 95% Cl: 1.24–9.34), and primary BSI (OR 3.29, 95% Cl: 1.24–9.34) were associated with the presence of an ESBL strain (Table 2). Antimicrobial use during current admission (OR 4.31, 95% Cl: 2.10–9.38) and heart disease (OR 4.20, Cl 2.07–8.81) remained as statistically significant predictors in the multivariable modeling (multivariable Model A). The effect estimates for these two factors, i.e., antimicrobial use during current admission and heart disease, in multivariate Model B, which included both significant predictors from the variables’ statistical selection process and those suggested in the literature, were 5.63 (95% Cl 2.10–17.00) and 4.72 (95% Cl: 2.23–10.50), respectively.

Antibiotic use during current admission (*p* < 0.01), respiratory infection, i.e., pneumonia (*p* = 0.03), and recent history of surgery (*p* = 0.04) were more prevalent among patients with CR *Klebsiella pneumoniae* BSI than among those with non-CR *Klebsiella pneumoniae* BSI (Table 3). A multivariable analysis was not applied because of the small number of patients in the carbapenem group (*n* = 13). Between BSIs caused by ESBL (*n* = 61) and CR strains (*n* = 13), there were no significant differences (Table 4).

## 4. Discussion

Bloodstream infections due to resistant *Klebsiella pneumoniae* pose a significant problem as ESBL and CR strains are becoming more frequent, especially in healthcare facilities, and they are associated with significant mortality. Between 2014 and 2018, at the Nicosia General Hospital in Cyprus, we found that approximately one-third of the 175 BSIs caused by *Klebsiella pneumoniae* were due to ESBL strains and 7.4% were due to CR strains. According to the annual report of the EARS-Net 2019 [18], the European average antimicrobial resistance to third-generation cephalosporin from *Klebsiella pneumoniae* strains during the period 2015–2019 was 31.34%, and the average resistance to carbapenem was 7.34%. Also, between 2017 and 2021, the average antimicrobial resistance to third-generation cephalosporins in Europe was 32.5%, and the average resistance to carbapenems was 8.86% [19]. Our results confirmed the high rates of *Klebsiella pneumoniae* resistance to third-generation cephalosporins and carbapenems. Data from these networks also show that Southern and Southeastern European countries have a higher percentage of resistance compared with other European countries [20,21,22]. According to the Australian Group on Antimicrobial Resistance (AGAR) [23], which included antimicrobial resistance data from Gram-negative strains, *Klebsiella pneumoniae* antimicrobial resistance to third-generation cephalosporins was only 8.7%, highlighting differences among the rates of antimicrobial resistance in different parts of the world.

BSI due to resistant *Klebsiella pneumoniae* strains was associated with antibiotic use during current or previous hospitalization, a factor that was also mentioned in the study by Mathers AJ et al. [24] and in the systematic review by Longshaw et al. [25], which investigated risk factors for carbapenem-resistant Gram-negative bacterial infections. These findings highlight the need to implement and maintain antimicrobial stewardship programs and to reduce the overuse and misuse of certain classes of antibiotics. Moreover, antibiotic use in the previous three months was associated with increased rates of resistant *Klebsiella pneumoniae* strains in a publication by Nguyen et al. [26]. In our study, antibiotic use in the previous three months was found in almost half of bloodstream infections due to ESBL strains and in 69.2% of BSIs due to CR strains. 

Co-morbidities like diabetes mellitus, pulmonary disease, heart disease, malignancy, autoimmune disease, hepatic and kidney disease, alcohol use, and smoking were investigated as risk factors for BSI due to *Klebsiella pneumoniae* strains. In our study, patients with heart disease (mainly heart failure and coronary artery disease) had a higher risk of *Klebsiella pneumoniae* ESBL bloodstream infection. Heart disease is not a common risk factor for BSI due to *Klebsiella pneumoniae* [27,28,29]. In one prospective, multicenter study [30], which investigated the epidemiology and risk factors for community-onset bloodstream infections caused by ESBL strains, it was found that heart failure was the only independent risk factor for BSI infection due to the ESBL strain. 

In our study, diabetes mellitus was not a significant factor for *Klebsiella pneumoniae* ESBL bloodstream infection at the 5% level. However, more than half of patients with *Klebsiella pneumoniae* ESBL bloodstream infection had a history of diabetes mellitus, and the effect of diabetes mellitus was marginally non-significant in our analysis. Moreover, diabetes mellitus was found to be a significant factor for ESBL bloodstream infection in the studies by Isendahl et al. [31] and Hansen et al. [32]. Poorly controlled diabetes mellitus is also a major risk factor for *Klebsiella pneumoniae* invasive disease, and the mortality rates range from 4% to 11% [33]. These findings highlight how important it is for hyperglycemia to be controlled during hospitalization, as uncontrolled diabetes is associated with increased mortality [34,35,36].

CR strains in our study occurred more often in patients with recent surgery or hospital-associated pneumonia, but, because of the small sample size, further statistical analysis was not possible. Transmission of CR strains is predominantly nosocomial and, in some countries, *Klebsiella pneumoniae* CR strains have an increasing prevalence among hospital-acquired pathogens [37,38,39]. BSIs due to CR strains can prolong the length of hospitalization and significantly increase patient mortality. The reported mortality rate of BSI due to multi-resistant *Klebsiella pneumoniae* varies from 15% to 79% [40,41,42,43]. The mortality associated with ESBL strains is lower than with CR strains (26). Evidently, the high mortality in patients with BSI caused by CR strains, especially in ICU patients, underscores the significance of implementing rigorous infection control measures [44,45].

## 5. Conclusions

Previous antimicrobial use was associated with bloodstream infections caused by resistant *Klebsiella pneumoniae* strains in the largest tertiary care hospital in Cyprus. The results are in line with data from other European countries, contribute to current evidence on the clinical impact of serious infections caused by multidrug-resistant strains, and highlight the need to act upon the established threat of antimicrobial resistance worldwide. The implementation of antimicrobial stewardship programs and infection control programs is imperative to reduce antimicrobial resistance rates, improve patient outcomes, and limit healthcare costs as well as the adverse ecological consequences of antimicrobial overconsumption.

## Figures and Tables

**Table 1:** Comparison of patients’ variables between those with BSI * due to ESBL * and non-ESBL strains *.

Variable	Non ESBL (*n* = 101)	ESBL (*n* = 61)	*p*-Value *
Age (mean ± SD *)	61.09 ± 23.49	69.11 ± 13.87	0.02
Sex (male)	27 (26.73%)	27 (44.26%)	0.03
Antibiotic use in previous 3 months (yes)	41 (40.59%)	34 (55.74%)	0.07
Antibiotic use during hospitalization (yes)	43 (42.57%)	45 (73.77%)	<0.01
Nosocomial infection (yes)	64 (63.36%)	44 (72.13%)	0.30
Diabetes mellitus (yes)	39 (38.61%)	33 (54.09%)	0.07
Pulmonary disease (yes)	20 (19.8%)	17 (27.87%)	0.25
Heart diseases (yes)	34 (33.66%)	41 (67.21%)	<0.01
Cancer (yes)	23 (22.77%)	11 (18.03%)	0.55
Autoimmune disease (yes)	2 (1.98%)	3 (4.92%)	0.36
Hepatic disease (yes)	9 (8.91%)	3 (4.91%)	0.53
End-stage renal disease dialysis (yes)	18 (17.82%)	7 (11.47%)	0.37
Alcohol use (yes)	6 (5.94%)	8 (13.11%)	0.15
Smoking (yes)	21 (20.8%)	12 (19.67%)	1.00
Hospitalized (yes)	54 (53.46%)	42 (68.85%)	0.07
Hospitalized ICU * (yes)	7 (6.93%)	12 (19.67%)	0.02
Prosthetics (yes)	28 (27.72%)	26 (45.90%)	0.06
Surgery (yes)	37 (36.60%)	25 (41.00%)	0.62
Outcome in 28 days (discharge)	57 (56.43%)	41 (67.21%)	1.00
Pneumonia (yes)	27 (26.73%)	22 (36.06%)	0.22
Urinary tract infection (yes)	32 (31.68%)	26 (42.62%)	0.18
Bloodstream infection (yes)	7 (6.93%)	12 (19.67%)	0.02
Intravenous lines (yes)	5 (4.95%)	3 (4.91%)	1.00
Intrabdominal infections (yes)	6 (5.94%)	3 (4.91%)	1.00

* BSI: bloodstream infection, * ESBL: extended-spectrum beta-lactamase, * non-ESBL: non-extended-spectrum beta-lactamase, * ICU: intensive care unit, * *p*-values are from chi-squared tests except age (*t*-test), * SD: standard deviation.

**Table 2:** Logistic regression analyses on risk factors for the detection of *Klebsiella pneumoniae* ESBL * strain among *Klebsiella pneumoniae* BSI * patients.

ESBL	Univariable Model			Multivariable Model A			Multivariable Model B		
Variables	OR *	CI *	*p*-Value *	OR	CI	*p*-Value	OR	CI	*p*-Value
Age	1.02	**1.00–1.04**	**0.02**						
Sex (male versus (vs.) female)	2.18	**1.12–4.28**	**0.02**				1.69	0.77–3.72	0.19
Antibiotic use in previous 3 months (yes vs. no)	1.84	0.97–3.53	0.06						
Antibiotic during hospitalization (yes vs. no)	4.05	**2.04–8.39**	**<0.01**	**4.31**	**2.10–9.38**	**<0.01**	**5.63**	**2.10–17.00**	**<0.01**
Diabetes mellitus (yes vs. no)	1.87	0.99–3.59	0.06						
Pulmonary disease (yes vs. no)	1.56	0.74–3.3	0.24						
Heart disease (yes vs. no)	4.04	**2.08–8.06**	**<0.01**	**4.20**	**2.07–8.81**	**<0.01**	**4.72**	**2.23–10.50**	**<0.01**
Cancer (yes vs. no)	0.75	0.32–1.63	0.47						
Autoimmune disease (yes vs. no)	2.56	0.41–19.87	0.31						
Hepatic disease (yes vs. no)	0.53	0.11–1.86	0.35						
End-stage renal disease dialysis (yes vs. no)	0.60	0.22–1.47	0.28						
Alcohol (yes vs. no)	2.39	0.79–7.61	0.12						
Smoking (yes vs. no)	0.93	0.41–2.04	0.86						
Hospitalized (yes vs. no)	1.92	**0.99–3.81**	**0.05**				0.96	0.40–22.40	0.92
Hospitalized ICU * (yes vs. no)	3.29	**1.24–9.34**	**0.02**				1.50	0.50–4.73	0.48
Surgery (yes vs. no)	1.20	0.62–2.31	0.58						
Prosthetics (yes vs. no)	1.94	0.99–3.80	0.05						
Outcome (discharge)	1.03	0.47–2.30	0.95						
Pneumonia (yes vs. no)	1.55	0.78–3.07	0.21						
Urinary tract infection (yes vs. no)	1.60	0.83–3.10	0.16						
Bloodstream infection (yes vs. no)	3.29	**1.24–9.34**	**0.02**						
Intravenous lines (yes vs. no)	0.99	0.20–4.20	0.99						
Intra-abdominal infections (yes vs. no)	0.82	0.17–3.23	0.78						
Hospital-acquired infection (yes vs. no)	1.50	0.76–3.03	0.25				0.43	0.16–1.12	0.09

* BSI: bloodstream infection, * ESBL: extended-spectrum beta-lactamase, * ICU: intensive care unit, * *p*-values are from chi-squared tests except for age (*t*-test), * OR: Odds Ratio, * CI: Confidence Interval.

**Table 3:** Comparison between BSI * caused by CR * (*n* = 13) and non-CR * strain (*n* = 162).

Variable	Carbapenem (*n* = 13)	Non Carbapenem (*n* = 162)	*p*-Value *
Age (mean ± SD *)	67.85 ± 17.32	64.11 ± 20.73	0.53
Sex (male)	5 (38.40%)	54 (33.30%)	0.76
Antibiotic use in previous 3 months (yes)	9 (69.20%)	75 (46.30%)	0.15
Antibiotic during hospitalization (yes)	12 (92.30%)	87 (53.70%)	<0.01
Nosocomial infection (yes)	11 (84.60%)	108 (66.70%)	0.23
Pneumonia (yes)	8 (61.50%)	49 (30.20%)	0.03
Urinary tract infection (yes)	3 (23.10%)	58 (35.80%)	0.55
Bloodstream infection (yes)	1 (7.70%)	19 (11.70%)	1.00
Intravenous lines (yes)	1 (7.70%)	8 (4.90%)	0.51
Intraabdominal infection (yes)	0	9 (5.50%)	1.00
Diabetes mellitus (yes)	3 (30.80%)	72 (44.40%)	0.16
Pulmonary disease (yes)	5 (38.50%)	37 (22.80%)	0.31
Heart disease (yes)	7 (53.80%)	75 (46.30%)	0.77
Cancer (yes)	1 (7.70%)	34 (21.00%)	0.48
Autoimmune disease (yes)	0	5 (3.10%)	1.00
Hepatic disease (yes)	0	12 (7.40%)	1.00
End-stage renal disease dialysis (yes)	1 (7.70%)	25 (15.40%)	0.69
Alcohol (yes)	1 (7.70%)	14 (8.60%)	1.00
Smoking (yes)	2 (15.40%)	33 (20.40%)	1.00
Hospitalized (yes)	10 (77.00%)	96 (59.30%)	0.25
Hospitalized ICU * (yes)	4 (30.80%)	19 (11.70%)	0.07
Surgery (yes)	9 (69.20%)	62 (38.30%)	0.04
Prosthetics (yes)	8 (61.50%)	54 (33.30%)	0.07
Outcome (discharge)	10 (76.90%)	98 (60.50%)	1.00

* BSI: bloodstream infection, * CR: carbapenem-resistant, * non-CR: non-carbapenem-resistant, * ICU: intensive care unit, * *p*-values are from chi-squared tests except for age (*t*-test), * SD: standard deviation.

**Table 4:** Comparison between BSI * CR * strain (*n* = 13) and ESBL * strain (*n* = 61).

Variable	Carbapenem (*n* = 13)	ESBL (*n* = 61)	*p*-Value *
Age (mean ± SD *)	67.85 ± 17.32	69.11 ± 13.87	0.78
Sex (male)	5 (38.50%)	27 (44.30%)	0.79
Antibiotic use in previous 3 months (yes)	9 (69.20%)	34 (55.70%)	0.54
Antibiotic during hospitalization(yes)	12 (92.30%)	45 (73.80%)	0.27
Nosocomial infection (yes)	11 (84.60%)	44 (72.10%)	0.49
Pneumonia (yes)	8 (61.50%)	22 (36.10%)	0.12
Urinary tract infection (yes)	3 (23.10%)	26 (42.60%)	0.23
Bloodstream infection (yes)	1 (7.80%)	12 (19.70%)	0.44
Intravenous lines (yes)	1 (7.80%)	3 (4.90%)	0.55
Intraabdominal infection (yes)	0	3 (4.90%)	1.00
Diabetes mellitus (yes)	3 (23.10%)	33 (54.10%)	0.07
Pulmonary disease (yes)	5 (38.50%)	17 (27.90%)	0.51
Heart disease (yes)	7 (53.80%)	41 (67.20%)	0.36
Cancer (yes)	1 (7.80%)	11 (18.00%)	0.68
Autoimmune disease (yes)	0	3 (4.90%)	1.00
Hepatic disease (yes)	0	3 (4.90%)	1.00
End-stage renal disease dialysis (yes)	1 (7.80%)	7 (11.50%)	1.00
Alcohol (yes)	1 (7.80%)	8 (13.10%)	1.00
Smoking (yes)	2 (15.40%)	12 (19.70%)	1.00
Hospitalized (yes)	10 (76.90%)	42 (68.90%)	0.74
Hospitalized ICU * (yes)	4 (30.80%)	12 (19.70%)	0.46
Surgery (yes)	9 (69.20%)	25 (41.00%)	0.07
Prosthetics (yes)	8 (61.50%)	26 (42.60%)	0.24
Outcome (discharge)	10 (76.90%)	41 (67.20%)	1.00

* BSI: bloodstream infection, * ESBL: extended-spectrum beta-lactamase, * ICU: intensive care unit, * *p*-values are from chi-squared tests except for age (*t*-test), * SD: standard deviation.

## Data Availability

Data are available upon request to the corresponding authors.

## Supplementary Materials

The following supporting information can be downloaded at: https://www.mdpi.com/article/10.3390/pathogens12111277/s1, R script analyses code.
