# Supplementary material for: Factors Associated with Extended-Spectrum β-Lactamases and Carbapenem-Resistant Klebsiella pneumoniae Bloodstream Infections: A Five-Year Retrospective Study
Source: Pathogens. 2023 Oct 25;12(11):1277. doi: 10.3390/pathogens12111277 (PMC10675166; doi:10.3390/pathogens12111277)
Supplement: Supplementary file 1 [file pathogens-12-01277-s001.zip › pathogens-2649820-supplementary.pdf]

R-SCRIPT CODE, ESBL-CR ANALYSIS

```
> kpc4 <- read.delim("C:/Users/panagiotis/Desktop/KPC/kpc4.txt", str
ingsAsFactors=FALSE)
>   View(kpc4)
> kpc4$AGE <- as.numeric(kpc3$AGE)
> kpc4$SEX <- as.factor(kpc4$SEX)
> kpc4$ANTIB.BEF.3MONTH <- as.factor(kpc4$ANTIB.BEF.3MONTH)
> kpc4$ANTIB.DUR.HOSPITALIZASION <- as.factor(kpc4$ANTIB.DUR.HOSPITA
LIZASION)
> kpc4$CAR.AN <- as.factor(kpc4$CAR.AN)
> kpc4$ESBL <- as.factor(kpc4$ESBL)
> kpc4$KPC <- as.factor(kpc4$KPC)
> kpc4$DM <- as.factor(kpc4$DM)
> kpc4$PULMON <- as.factor(kpc4$PULMON)
> kpc4$CARDIO <- as.factor(kpc4$CARDIO)
> kpc4$CANCER <- as.factor(kpc4$CANCER)
> kpc4$AUTOIM <- as.factor(kpc4$AUTOIM)
> kpc4$HEPAT <- as.factor(kpc4$HEPAT)
> kpc4$DIALISIS <- as.factor(kpc4$DIALISIS)
> kpc4$ALCOHOL <- as.factor(kpc4$ALCOHOL)
> kpc4$SMOKING <- as.factor(kpc4$SMOKING)
> kpc4$HOSPITALIZED <- as.factor(kpc4$HOSPITALIZED)
> kpc4$HOSPIT.ICU <- as.factor(kpc4$HOSPIT.ICU)
> kpc4$SURGERY <- as.factor(kpc4$SURGERY)
> kpc4$PROSTHETICS <- as.factor(kpc4$PROSTHETICS)
> kpc4$OUTCOME <- as.factor(kpc4$OUTCOME)
> kpc4$PNEUMONIA <- as.factor(kpc4$PNEUMONIA)
> kpc4$UTI <- as.factor(kpc4$UTI)
> kpc4$BLOOODSTREEM <- as.factor(kpc4$BLOOODSTREEM)
> kpc4$LINES <- as.factor(kpc4$LINES)
> kpc4$INTRAABDOMINAL <- as.factor(kpc4$INTRAABDOMINAL)
> lapply(c("AGE","SEX","ANTIB.BEF.3MONTH","ANTIB.DUR.HOSPITALIZASION
","PNEUMONIA", "UTI", "BLOOODSTREEM", "LINES", "INTRAABDOMINAL", "DM
", "PULMON", "CARDIO", "CANCER", "AUTOIM", "HEPAT", "DIALISIS", "ALC
OHOL", "SMOKING", "HOSPITALIZED", "HOSPIT.ICU", "OUTCOME", "SURGERY"
, "PROSTHETICS","HAC"),
+        function(x) {
+            formula <- as.formula(paste("ESBL ~", x))
+            res <- glm(formula, data = kpc4, family = binomial())
+            print(summary(res))
+            print(cbind(exp(coef(res)), exp(confint(res))))
+            anova(res)
+        })
> Sexesbl.tb<-with(kpc4, table(SEX, ESBL))
> CrossTable(Sexesbl.tb,expected=F,prop.c=F,prop.t=F,prop.chisq=F, f
isher=T)
> Antibef3mesbl.tb<-with(kpc4, table(ANTIB.BEF.3MONTH, ESBL))
> CrossTable(Antibef3mesbl.tb,expected=F,prop.c=F,prop.t=F,prop.chis
q=F, fisher=T)
  Antdurhospesbl.tb<-with(kpc4, table(ANTIB.DUR.HOSPITALIZASION, ESB
L))
>  CrossTable(Antdurhospesbl.tb,expected=F,prop.c=F,prop.t=F,prop.ch
isq=F, fisher=T)
> Dmesbl.tb<-with(kpc4, table(DM, ESBL))
> CrossTable(Dmesbl.tb,expected=F,prop.c=F,prop.t=F,prop.chisq=F, fi
sher=T)

> Pulmonesbl.tb<-with(kpc4, table(PULMON, ESBL))
> CrossTable(Pulmonesbl.tb,expected=F,prop.c=F,prop.t=F,prop.chisq=F
, fisher=T)
> Cardioesbl.tb<-with(kpc4, table(CARDIO, ESBL))
> CrossTable(Cardioesbl.tb,expected=F,prop.c=F,prop.t=F,prop.chisq=F
, fisher=T)
> Canceresbl.tb<-with(kpc4, table(CANCER, ESBL))
> CrossTable(Canceresbl.tb,expected=F,prop.c=F,prop.t=F,prop.chisq=F
, fisher=T)
> Autoimesbl.tb<-with(kpc4, table(AUTOIM, ESBL))
```

```
> CrossTable(Autoimesbl.tb,expected=F,prop.c=F,prop.t=F,prop.chisq=F
, fisher=T)
> Hepatesbl.tb<-with(kpc4, table(HEPAT, ESBL))
> CrossTable(Hepatesbl.tb,expected=F,prop.c=F,prop.t=F,prop.chisq=F,
fisher=T)
> Dialisisesbl.tb<-with(kpc4, table(DIALISIS, ESBL))
> CrossTable(Dialisisesbl.tb,expected=F,prop.c=F,prop.t=F,prop.chisq
=F, fisher=T)
> Alcoholesbl.tb<-with(kpc4, table(ALCOHOL, ESBL))
> CrossTable(Alcoholesbl.tb,expected=F,prop.c=F,prop.t=F,prop.chisq=
F, fisher=T)
> Hospitalizedesbl.tb<-with(kpc4, table(HOSPITALIZED, ESBL))
> CrossTable(Hospitalizedesbl.tb,expected=F,prop.c=F,prop.t=F,prop.c
hisq=F, fisher=T)

> Hospiticuesbl.tb<-with(kpc4, table(HOSPIT.ICU, ESBL))
> CrossTable(Hospiticuesbl.tb,expected=F,prop.c=F,prop.t=F,prop.chis
q=F, fisher=T)

> Prostheticsesbl.tb<-with(kpc4, table(PROSTHETICS, ESBL))
> CrossTable(Prostheticsesbl.tb,expected=F,prop.c=F,prop.t=F,prop.ch
isq=F, fisher=T)
> Outcomeesbl.tb<-with(kpc4, table(OUTCOME, ESBL))
> CrossTable(Outcomeesbl.tb,expected=F,prop.c=F,prop.t=F,prop.chisq=
F, fisher=T)

> Pneumoniaesbl.tb<-with(kpc4, table(PNEUMONIA, ESBL))
> CrossTable(Pneumoniaesbl.tb,expected=F,prop.c=F,prop.t=F,prop.chis
q=F, fisher=T)
> Utiesbl.tb<-with(kpc4, table(UTI, ESBL))
> CrossTable(Utiesbl.tb,expected=F,prop.c=F,prop.t=F,prop.chisq=F, f
isher=T)
> Blooodstreemesbl.tb<-with(kpc4, table(BLOOODSTREEM, ESBL))
> CrossTable(Blooodstreemesbl.tb,expected=F,prop.c=F,prop.t=F,prop.c
hisq=F, fisher=T)

  Linesesbl.tb<-with(kpc4, table(LINES, ESBL))
>  CrossTable(Linesesbl.tb,expected=F,prop.c=F,prop.t=F,prop.chisq=F
, fisher=T)
>
>  Blooodstreemesbl.tb<-with(kpc4, table(BLOOODSTREEM, ESBL))
>  CrossTable(Blooodstreemesbl.tb,expected=F,prop.c=F,prop.t=F,prop.
chisq=F, fisher=T)
> Hacesbl.tb<-with(kpc4, table(HAC, ESBL))
> CrossTable(Hacesbl.tb,expected=F,prop.c=F,prop.t=F,prop.chisq=F, f
isher=T)

> descriptives<-function(x){
+     cat("Number of observations=", length(x),"\n")
+     cat("Mean=", round(mean(x),digits=2),"\n")
+     cat("Std. Deviation=",round(sd(x),digits=2),"\n")
+     cat("Variance=", round(var(x),digits=2), "\n")
+     cat("Conf. Interval->", round(CI(x, ci = 0.95),digits=2),"\n")
+     cat("Median=", round(median(x),digits=2),"\n")
+     cat("Quantiles->", round(quantile(x),digits=2),"\n")
+     cat("Inter-quartile Range=",round(IQR(x),digits=2),"\n")
+     cat("Range=", round(max(x)-min(x),digits=2),"\n")
+     cat("Skewness=", round(skew(x),digits=3),"\n")
+     cat("Kurtosis=", round(kurt(x),digits=3),"\n")
+     cat("Missing values(NA)=", sum(is.na(x)),"\n")
+ }
> with(kpc4, descriptives(AGE))
> with(kpc4, descriptives(AGE [ESBL=="0"]))
> with(kpc4, descriptives(AGE [ESBL=="1"]))
> library(nortest)
> with(kpc4, t.test(AGE~ESBL,var.equal=T))

library(tcltk)
> library(lattice)
```

```
> multilogesbl <- glm(ESBL ~ AGE + SEX + ANTIB.BEF.3MONTH + ANTIB.DU
R.HOSPITALIZASION + DM + PULMON + CARDIO + HOSPITALIZED + HOSPIT.ICU
+ PROSTHETICS + BLOOODSTREEM, data = kpc4,
+                   family = binomial())
> multilogesbl
> summary(multilogesbl)

>  cbind(exp(coef(multilogesbl)), exp(confint(multilogesbl)))
> multilogesbl1 <- glm(ESBL ~ AGE  + ANTIB.DUR.HOSPITALIZASION +  CA
RDIO, data = kpc4,
+                   family = binomial())
> summary(multilogesbl1

> multilogesbl2 <- glm(ESBL ~ SEX  + ANTIB.DUR.HOSPITALIZASION +  CA
RDIO, data = kpc4,
+                   family = binomial())
> summary(multilogesbl2)
> multilogesbl3 <- glm(ESBL ~ ANTIB.BEF.3MONTH  + ANTIB.DUR.HOSPITAL
IZASION +  CARDIO, data = kpc4,
+                   family = binomial())
> summary(multilogesbl3)
> multilogesbl4 <- glm(ESBL ~ DM  + ANTIB.DUR.HOSPITALIZASION +  CAR
DIO, data = kpc4,
+                   family = binomial())
> summary(multilogesbl4)

> multilogesbl5 <- glm(ESBL ~ PULMON  + ANTIB.DUR.HOSPITALIZASION +
CARDIO, data = kpc4,
+                   family = binomial())
> summary(multilogesbl5)


> multilogesbl6 <- glm(ESBL ~ CANCER  + ANTIB.DUR.HOSPITALIZASION +
CARDIO, data = kpc4,
+                   family = binomial())
> summary(multilogesbl6)

> multilogesbl7 <- glm(ESBL ~ DIALISIS  + ANTIB.DUR.HOSPITALIZASION
+  CARDIO, data = kpc4,
+                   family = binomial())
> summary(multilogesbl7)
> multilogesb8 <- glm(ESBL ~ HOSPITALIZED  + ANTIB.DUR.HOSPITALIZASI
ON +  CARDIO, data = kpc4,
+                   family = binomial())
> summary(multilogesb8)
> multilogesbL9 <- glm(ESBL ~ HOSPIT.ICU  + ANTIB.DUR.HOSPITALIZASIO
N +  CARDIO, data = kpc4,
+                   family = binomial())
> summary(multilogesbL9

> multilogesbL10 <- glm(ESBL ~ SURGERY  + ANTIB.DUR.HOSPITALIZASION
+  CARDIO, data = kpc4,
+                   family = binomial())
> summary(multilogesbL10)

> multilogesbL11 <- glm(ESBL ~ PROSTHETICS  + ANTIB.DUR.HOSPITALIZAS
ION +  CARDIO, data = kpc4,
+                   family = binomial())
> summary(multilogesbL11)

> multilogesbL12 <- glm(ESBL ~ PNEUMONIA  + ANTIB.DUR.HOSPITALIZASIO
N +  CARDIO, data = kpc4,
+                   family = binomial())
> summary(multilogesbL12)
> multilogesbL13 <- glm(ESBL ~ UTI  + ANTIB.DUR.HOSPITALIZASION +  C
ARDIO, data = kpc4,
+                   family = binomial())
> summary(multilogesbL13)
```

```
> multilogesbL14 <- glm(ESBL ~ BLOOODSTREEM  + ANTIB.DUR.HOSPITALIZA
SION +  CARDIO, data = kpc4,
+                  family = binomial())
> summary(multilogesbL14)
> multilogEsbl <- glm(ESBL ~ AGE + SEX + HAC + ANTIB.BEF.3MONTH + AN
TIB.DUR.HOSPITALIZASION + DM + PULMON + CARDIO + HOSPITALIZED + HOSP
IT.ICU + PROSTHETICS + BLOOODSTREEM, data = kpc4,
+                      family = binomial())
> summary(multilogEsbl)
> multilogEsbl1 <- glm(ESBL ~ HAC  + ANTIB.DUR.HOSPITALIZASION +  CA
RDIO, data = kpc4,
+                       family = binomial())
> summary(multilogEsbl1)

> multilogESBL <- glm(ESBL ~ HAC + HOSPITALIZED + HOSPIT.ICU + SEX
+ ANTIB.DUR.HOSPITALIZASION +  CARDIO, data = kpc4,
+                       family = binomial())
> summary(multilogESBL)

> multilogEsbl <- glm(ESBL ~ AGE + SEX + HAC + ANTIB.BEF.3MONTH + AN
TIB.DUR.HOSPITALIZASION + DM + PULMON + CARDIO + HOSPITALIZED + HOSP
IT.ICU + PROSTHETICS + BLOOODSTREEM, data = kpc4,
+                      family = binomial())
> summary(multilogEsbl)

> multilogEsbl1 <- glm(ESBL ~ HAC  + ANTIB.DUR.HOSPITALIZASION +  CA
RDIO, data = kpc4,
+                       family = binomial())
> summary(multilogEsbl1)

> multilogESBL <- glm(ESBL ~ HAC + HOSPITALIZED + HOSPIT.ICU + SEX
+ ANTIB.DUR.HOSPITALIZASION +  CARDIO, data = kpc4,
+                       family = binomial())
> summary(multilogESBL)
> cbind(exp(coef(multilogESBL)), exp(confint(multilogESBL)))
> multilogesbL15 <- glm(ESBL ~ ANTIB.DUR.HOSPITALIZASION +  CARDIO,
data = kpc4,
+                        family = binomial())
> summary(multilogesbL15)

> cbind(exp(coef(multilogesbL15)), exp(confint(multilogesbL15)))
lapply(c(("AGE","SEX","DURATION.ICU","ANTIB.BEF.3MONTH","ANTIB.DUR.H
OSPITALIZASION","TIG.AN","ESBL", "KPC", "DM", "PULMON", "CARDIO", "C
ANCER", "AUTOIM", "HEPAT", "DIALISIS", "ALCOHOL", "SMOKING", "HOSPIT
ALIZED", "HOSPIT.ICU", "SURGERY", "PROSTHETICS", "OUTCOME""AMP.AN",
"PNEUMONIA", "UTI", "BLOOODSTREEM", "LINES", "INTRAABDOMINAL", "CELU
LITIS", "CENTRAL.NERVOUS","HAC", "BLOOD", "URINE", "SPUTUM.BAL", "CS
F", "FORIA", "TYPE.INF","AGE","OUTCOME"),
+        function(x) {
+            formula <- as.formula(paste("CAR.AN ~", x))
+            res <- glm(formula, data = kpc2, family = binomial())
+            print(summary(res))
+            print(cbind(exp(coef(res)), exp(confint(res))))
+            anova(res)
+        })
sexkpc.tb<-with(kpc3, table(SEX, CAR.AN))
> CrossTable(sexkpc.tb,expected=F,prop.c=F,prop.t=F,prop.chisq=F, fi
sher=T)
> antibef3mkpc.tb<-with(kpc3, table(ANTIB.BEF.3MONTH, CAR.AN))
> CrossTable(antibef3mkpc.tb,expected=F,prop.c=F,prop.t=F,prop.chisq
=F, fisher=T)

> antidurhoskpc.tb<-with(kpc3, table(ANTIB.DUR.HOSPITALIZASION, CAR.
AN))
> CrossTable(antidurhoskpc.tb,expected=F,prop.c=F,prop.t=F,prop.chis
q=F, fisher=T)

> dmkpc.tb<-with(kpc3, table(DM, CAR.AN))
```

```
> CrossTable(dmkpc.tb,expected=F,prop.c=F,prop.t=F,prop.chisq=F, fis
her=T)
> sputumbalkpc.tb<-with(kpc3, table(SPUTUM.BAL, CAR.AN))
> CrossTable(sputumbalkpc.tb,expected=F,prop.c=F,prop.t=F,prop.chisq
=F, fisher=T)

> pulmonkpc.tb<-with(kpc3, table(PULMON, CAR.AN))
> CrossTable(pulmonkpc.tb,expected=F,prop.c=F,prop.t=F,prop.chisq=F,
fisher=T)

> cardiokpc.tb<-with(kpc3, table(CARDIO, CAR.AN))
> CrossTable(cardiokpc.tb,expected=F,prop.c=F,prop.t=F,prop.chisq=F,
fisher=T)

> cancerkpc.tb<-with(kpc3, table(CANCER, CAR.AN))
> CrossTable(cancerkpc.tb,expected=F,prop.c=F,prop.t=F,prop.chisq=F,
fisher=T)
> autoimkpc.tb<-with(kpc3, table(AUTOIM, CAR.AN))
> CrossTable(autoimkpc.tb,expected=F,prop.c=F,prop.t=F,prop.chisq=F,
fisher=T)

> hospitalizedkpc.tb<-with(kpc3, table(HOSPITALIZED, CAR.AN))
> CrossTable(hospitalizedkpc.tb,expected=F,prop.c=F,prop.t=F,prop.ch
isq=F, fisher=T)

> hospiticukpc.tb<-with(kpc3, table(HOSPIT.ICU, CAR.AN))
> CrossTable(hospiticukpc.tb,expected=F,prop.c=F,prop.t=F,prop.chisq
=F, fisher=T)

> surgerykpc.tb<-with(kpc3, table(SURGERY, CAR.AN))
> CrossTable(surgerykpc.tb,expected=F,prop.c=F,prop.t=F,prop.chisq=F
, fisher=T)
> prostheticskpc.tb<-with(kpc3, table(PROSTHETICS, CAR.AN))
> CrossTable(prostheticskpc.tb,expected=F,prop.c=F,prop.t=F,prop.chi
sq=F, fisher=T)
> outcomekpc.tb<-with(kpc3, table(OUTCOME, CAR.AN))
> CrossTable(outcomekpc.tb,expected=F,prop.c=F,prop.t=F,prop.chisq=F
, fisher=T)
> pneumoniakpc.tb<-with(kpc3, table(PNEUMONIA, CAR.AN))
> CrossTable(pneumoniakpc.tb,expected=F,prop.c=F,prop.t=F,prop.chisq
=F, fisher=T)

> with(kpc5, table(ESBLCAR))

> with(kpc5, table(ESBLCAR, OUTCOME))

>lapply(c("AGE","SEX","ANTIB.BEF.3MONTH","ANTIB.DUR.HOSPITALIZASION"
,"PNEUMONIA", "UTI", "BLOOODSTREEM", "LINES", "INTRAABDOMINAL",
"DM", "PULMON", "CARDIO", "CANCER", "AUTOIM", "HEPAT", "DIALISIS",
"ALCOHOL", "SMOKING", "HOSPITALIZED", "HOSPIT.ICU", "OUTCOME",
"SURGERY", "PROSTHETICS", "HAC"),
+ function(x) {
+ formula <- as.formula(paste("ESBLCAR ~", x))
+ res <- glm(formula, data = kpc5, family = binomial())
+ print(summary(res))
+ print(cbind(exp(coef(res)), exp(confint(res))))
+ anova(res)
+ })

> with(kpc5, descriptives(AGE))
> with(kpc5, descriptives(AGE [ESBLCAR=="0"]))
> with(kpc5, descriptives(AGE [ESBLCAR=="1"]))
> library(nortest)
> with(kpc5, t.test(AGE~ESBLCAR,var.equal=T))

> with(kpc5, table(ESBLCAR))
> SURGERYEC.tb<-with(kpc5, table(SURGERY, ESBLCAR))
> CrossTable(SURGERYEC.tb,expected=F,prop.c=F,prop.t=F,prop.chisq=F,
fisher=T)
```

```
> PROSTHETICEC.tb<-with(kpc5, table(PROSTHETICS, ESBLCAR))
> CrossTable(PROSTHETICEC.tb,expected=F,prop.c=F,prop.t=F,prop.chisq
=F, fisher=T)
> OUTCOMEEC.tb<-with(kpc5, table(OUTCOME, ESBLCAR))
> CrossTable(OUTCOMEEC.tb,expected=F,prop.c=F,prop.t=F,prop.chisq=F,
fisher=T)
> SEXEC.tb<-with(kpc5, table(SEX, ESBLCAR))
> CrossTable(SEXEC.tb,expected=F,prop.c=F,prop.t=F,prop.chisq=F, fis
her=T)
> ANTB3MEC.tb<-with(kpc5, table(ANTIB.BEF.3MONTH, ESBLCAR))
> CrossTable(ANTB3MEC.tb,expected=F,prop.c=F,prop.t=F,prop.chisq=F,
fisher=T)
> ANDHEC.tb<-with(kpc5, table(ANTIB.DUR.HOSPITALIZASION, ESBLCAR))
> CrossTable(ANDHEC.tb,expected=F,prop.c=F,prop.t=F,prop.chisq=F, fi
sher=T)
> HACEC.tb<-with(kpc5, table(HAC, ESBLCAR))
> CrossTable(HACEC.tb,expected=F,prop.c=F,prop.t=F,prop.chisq=F, fis
her=T)
> PNEUMONIAEC.tb<-with(kpc5, table(PNEUMONIA, ESBLCAR))
> CrossTable(PNEUMONIAEC.tb,expected=F,prop.c=F,prop.t=F,prop.chisq=
F, fisher=T)
> UTIEC.tb<-with(kpc5, table(UTI, ESBLCAR))
> CrossTable(UTIEC.tb,expected=F,prop.c=F,prop.t=F,prop.chisq=F, fis
her=T)
> BLOOEC.tb<-with(kpc5, table(BLOOODSTREEM, ESBLCAR))
> CrossTable(BLOOEC.tb,expected=F,prop.c=F,prop.t=F,prop.chisq=F, fi
sher=T)

 > LINESEC.tb<-with(kpc5, table(LINES, ESBLCAR))
> CrossTable(LINESEC.tb,expected=F,prop.c=F,prop.t=F,prop.chisq=F, f
isher=T)

> INTRAABDEC.tb<-with(kpc5, table(INTRAABDOMINAL, ESBLCAR))
> CrossTable(INTRAABDEC.tb,expected=F,prop.c=F,prop.t=F,prop.chisq=F
, fisher=T)
> DMEC.tb<-with(kpc5, table(DM, ESBLCAR))
> CrossTable(DMEC.tb,expected=F,prop.c=F,prop.t=F,prop.chisq=F, fish
er=T)
> PULMONEC.tb<-with(kpc5, table(PULMON, ESBLCAR))
>
> CrossTable(PULMONEC.tb,expected=F,prop.c=F,prop.t=F,prop.chisq=F,
fisher=T)
> CARDIOEC.tb<-with(kpc5, table(CARDIO, ESBLCAR))
>
> CrossTable(CARDIOEC.tb,expected=F,prop.c=F,prop.t=F,prop.chisq=F,
fisher=T)
> CANCEREC.tb<-with(kpc5, table(CANCER, ESBLCAR))
>
> CrossTable(CANCEREC.tb,expected=F,prop.c=F,prop.t=F,prop.chisq=F,
fisher=T)
> AUTOIMEC.tb<-with(kpc5, table(AUTOIM, ESBLCAR))
>
> CrossTable(AUTOIMEC.tb,expected=F,prop.c=F,prop.t=F,prop.chisq=F,
fisher=T)
> HEPATEC.tb<-with(kpc5, table(HEPAT, ESBLCAR))
> CrossTable(HEPATEC.tb,expected=F,prop.c=F,prop.t=F,prop.chisq=F, f
isher=T)
> DIALISISEC.tb<-with(kpc5, table(DIALISIS, ESBLCAR))
> CrossTable(DIALISISEC.tb,expected=F,prop.c=F,prop.t=F,prop.chisq=F
, fisher=T)
> ALCOHOLEC.tb<-with(kpc5, table(ALCOHOL, ESBLCAR))
> CrossTable(ALCOHOLEC.tb,expected=F,prop.c=F,prop.t=F,prop.chisq=F,
fisher=T)
> SMOKINGEC.tb<-with(kpc5, table(SMOKING, ESBLCAR))
> CrossTable(SMOKINGEC.tb,expected=F,prop.c=F,prop.t=F,prop.chisq=F,
fisher=T)
> SMOKINGEC.tb<-with(kpc5, table(SMOKING, ESBLCAR))
> CrossTable(SMOKINGEC.tb,expected=F,prop.c=F,prop.t=F,prop.chisq=F,
fisher=T)
```

```
> HOSPITICUEC.tb<-with(kpc5, table(HOSPIT.ICU, ESBLCAR))
> CrossTable(HOSPITICUEC.tb,expected=F,prop.c=F,prop.t=F,prop.chisq=
F, fisher=T)
```
